# CircRNA_100876 Is Upregulated in Gastric Cancer (GC) and Promotes the GC Cells’ Growth, Migration and Invasion via miR-665/YAP1 Signaling

**DOI:** 10.3389/fgene.2020.546275

**Published:** 2020-11-11

**Authors:** Xiaosheng Lin, Cong Huang, Zhian Chen, Huaiming Wang, Yongming Zeng

**Affiliations:** ^1^Department of Gastrointestinal Surgery, The First Affiliated Hospital of Shantou University Medical College, Shantou, China; ^2^Department of Ultrasound, The First Affiliated Hospital of Shantou University Medical College, Shantou, China; ^3^The First College of Clinical Medicine, Southern Medical University, Guangzhou, China

**Keywords:** gastric cancer, YAP1, cell metastasis, miR-665, circRNA_100876

## Abstract

The present study aimed to investigate the biological function and relative mechanisms of circRNA_100876 in gastric cancer (GC). To this end, quantitative real-time polymerase chain reaction (RT-qPCR) was performed to examine the expression of circRNA_100876 and miR-665 in GC tissues and cells, and circRNA_100876 expression was depleted by the transfection of circ_100876-targeting siRNAs. CCK-8, flow cytometry, and Transwell assays were applied to examine GC cell cycle distribution, proliferation, apoptosis, migration, and invasion abilities. Proteins related to apoptosis and epithelial-mesenchymal transition (EMT) were detected by western blotting. Luciferase reporter assays were conducted to verify the direct target site between circRNA_100876 and miR-665. Our study confirmed that circRNA_100876 was highly expressed in GC lesions compared with the adjacent normal tissues (*P* < 0.001). High circRNA_100876 expression was negatively associated with survival outcome (*P* = 0.000). Furthermore, the down-regulation of circRNA_100876 could inhibit GC cell proliferation, invasion, and migration by suppressing the EMT pathway. Further study suggested that circRNA_100876 could act as a competing endogenous RNA by sequestering miR-665, and luciferase activity assay indicated that circRNA_100876 could bind directly with miR-665. Moreover, we found that Yes-associated protein 1 (YAP1) was the downstream target gene of miR-665, miR-665 knockdown could up-regulate YAP1 expression in MKN45 cells, and YAP1 knockdown could inhibit MKN45 cell proliferation, migration and invasion. Therefore, we demonstrated that circRNA_100876 over-expression in GC could promote GC tumor growth, migration and invasion and exert its effects through miR-665/YAP1 signaling.

## Introduction

Gastric cancer (GC) is one of the most prevalent malignancy worldwide and is well known for its high morbidity and mortality. Currently, the incidence of GC continues to rise, especially in China, which accounts for about 50% of the cases worldwide (Strong et al., 2015). In recent years, immunotherapy and targeted therapy have achieved major breakthroughs, but the 5-year survival rate remains unsatisfactory (Shuyama et al., 2007; Lin et al., 2017). This is largely due to the highly limited understanding of the exact molecular mechanism responsible for the early occurrence and development of GC. Therefore, it is urgent and important to screen effective molecular markers, achieve early diagnosis and survival prediction for groups at high risk of GC, and further study the mechanism of GC occurrence and development (Khalil et al., 2009; Shih et al., 2017).

In recent years, studies have shown that endogenous RNAs, which are widely found in eukaryotes, such as microRNAs (miRNAs), long non-coding RNAs (lncRNAs), and circular RNAs (circRNAs), play an important regulatory role in various cellular physiological processes and may be involved in tumor formation and progression (Prensner and Chinnaiyan, 2011; Shi et al., 2013). Unlike lncRNAs, circRNAs lack 5′–3′ polarity and polyadenylated tails, instead having covalently closed loop structures which can resist RNA exonuclease and RNase R activity (Hansen et al., 2013a; Memczak et al., 2013), making circRNAs more stable than lncRNAs. Accumulating research has elucidated that circRNAs are widely involved in diverse physiological and pathological processes, especially tumor generation and development (Hansen et al., 2013b).

CircRNA_100876 is a novel competing endogenous RNA (ceRNA). Previous studies indicated that circRNA_100876 was involved in the regulation of matrix metallopeptidase 13 (MMP13) expression, and MMP13 had been identified as a significant regulators of cancer cell migration and invasion abilities via degrading extracellular matrix (Hsu et al., 2006; Liu et al., 2016). Therefore, circRNA_100876 might play an important role in the regulation of various cancer progression. To date, circRNA_100876 has been confirmed to be up-regulated in lung cancer cells and esophageal squamous cancer cells, and patients over-expressing circRNA_100876 tended to have a poor prognosis (Yao J.T. et al., 2017; Cao et al., 2018). However, the specific role of circRNA_100876 in GC progression is still not clear and needs further research.

Recently, numerous studies demonstrated that the circ_RNAs could directly inhibit the function of miRNAs via cavernous mechanism, and then exert cancer promotion or tumor suppression effect (Yao T. et al., 2017; Panda, 2018). For example, previous studies indicated that miRNA-665 (miR-665) was down-regulated in GC tissues, and it could serve as a tumor suppressor in GC progression (Wu et al., 2020; Zhang et al., 2020). Interestingly, based on the results of public database, miR-665 might be the downstream target gene of circRNA_100876, but the interaction between circRNA_100876 and miR-665 had not been explored in GC progression.

Yes-associated protein 1 (YAP1) is a transcriptional effector component of the Hippo pathway, which was involved in the regulation of cancer cell proliferation and apoptosis, especially in gastrointestinal cancer (Kang et al., 2011; Sabra et al., 2017; Zhang et al., 2018). For example, Kang et al. (2011) demonstrated that YAP1 exhibits oncogenic property in GC via activating the early-response gene pathway. However, whether YAP1 is the downstream target gene of circRNA_100876/miR-665 axis need further research (Kang et al., 2011).

In this study, we performed *in vitro* experiments to detect circRNA_100876 expression in GC tissues and its potential relationship with the clinicopathologic parameters. More importantly, we also evaluated GC cell proliferation, apoptosis, migration, and invasion after the down-regulation of circRNA_100876 in GC cells. Further investigation revealed that circRNA_100876 could serve as a ceRNA for miR-665) to regulate YAP1 expression.

## Materials and Methods

### Samples of GC Patients

Human GC samples and adjacent normal tissues were collected from 100 patients who had undergone GC surgical resection between 2014 and 2017. In addition, all pathological results of the tissue samples were determined by experienced pathologists. All patients voluntarily signed informed consent. After excision, the tissues were quickly frozen and stored at −80°C. Moreover, this study was approved by the Ethics Committee of Shantou University Medical College.

### Cell Culture

Normal human gastric cells (GES-1) and five human GC cell lines (BGC-823, SGC-7901, AGS, MKN45, and MGC-803) were purchased from ATCC (Shanghai, China). Subsequently, McCoy’s 5a Medium (Gibco, Grand Island, NY, United States) containing 10% fetal bovine serum (Australia origin, Gibco) was used for cell culturing. Finally, these cells were incubated in a humid environment with 5% CO_2_ at 37°C.

### RNA Transfection

To construct the circRNA_100876-down-regulated cell models, AGS and MKN45 cells were treated with 5 μg/mL polybrene and lentiviruses [multiplicity of infection (MOI) = 100]. Subsequently, puromycin was used to screen the stable circRNA_100876-down-regulated cell lines (siRNA-1 and siRNA-2 cells). The sequence is listed in Table 1.

**TABLE 1:** Sequences of siRNAs and primers used in the present research.

Name	Sequence (5′-3′)
Si-circRNA_100876-1	CAC GCT CCT ACA ATG TTG ATA
Si-circRNA_100876-2	CCA CGC TCC TAC AAT GTT GAT
Negative control	TTC TCC GAA CGT GTC ACG TTT
CircRNA_100876 forward	CTG GTG CAG TGG AAG CAG AG
CircRNA_100876 reverse	CGA CCC TCC ATT GCT CTT CT

### Quantitative Real-Time Polymerase Chain Reaction (RT-qPCR)

Firstly, the total RNA was extracted from the related tissues and cells with RNAiso Plus reagent (TaKaRa, Dalian, China). According to manufacturer’s instructions, the PrimeScript RT Master Mix (TaKaRa) was used to reverse transcribe 500 ng total RNA into cDNA. On the one hand, the expression level of circRNA_100876 were measured with the One Step SYBR^®^ PrimeScript^TM^ RT-PCR Kit II (Takara, Kusatsu, Japan) via RT-qPCR assays, and its expression levels was normalized with GAPDH. On the other hand, the expression level of miR-665 were measured with the TaqMan MicroRNA Assays Kit (Applied Biosystems, Carlsbad, CA, United States) via RT-qPCR assays, and its expression levels was normalized with U6. Furthermore, the 2^–ΔΔCt^ method was applied to evaluate fold changes.

### *In vitro* Cell Proliferation Assay

In this study, we used the Cell Counting Kit 8 (CCK-8, Dojindo, Kumamoto, Japan) assay to evaluate the cell proliferation ability. Firstly, the circRNA_100876-down-regulated GC cells were collected and cultured in 96-well plates. After 0, 24, 48, 72, and 96 h, 100 μL McCoy’s 5a Medium containing 10% CCK-8 was added to each well, and the cell viability was evaluated with a microplate reader (Bio-Rad, Hercules, CA, United States). For EDU assay, circRNA_100876-down-regulated GC cells were collected and planted in 96-well plates for 24 h, following by being washed with PBS and stained with EDU solutions (Ruibo, Guangzhou, China), then the cells proliferation abilities were evaluated with the fluorescence microscope based on the red fluorescence intensity.

### Flow Cytometric Analyses

According to the manufacturer’s protocol, the Annexin V-FITC/PI apoptosis detection kit (Keygen, Nanjing, China) was used to detect the cell apoptosis rate with the FACSCanto II flow cytometer (BD Biosciences). Subsequently, the cell cycle detection kit (Keygen) was applied for cell cycle analysis using the manufacturer’s protocol.

### Transwell Assay

Transwell chambers (0.8 μm; Corning Inc., Corning, NY, United States) with or without Matrigel coating (Corning) were used to detect the cell migration and invasion abilities, respectively. Then, 100 μL serum-free medium containing 3 × 10^4^ cells was added to the upper chambers, while 500 μL McCoy’s 5a Medium containing 20% FBS was added to the lower chambers. After 24 h, the cells were fixed and stained for digital imaging.

### Western Blot Analysis

RIPA Lysis buffer (Beyotime, Shanghai, China) was used to extract the total protein, and the BCA Protein Assay Kit (Thermo Fisher Scientific, Shanghai, China) was used to detect the protein concentration. Equivalent amounts of protein (20 μg) were separated on SDS-PAGE gels and transferred onto polyvinylidene fluoride (PVDF) membranes, which were collected and incubated with 10% BSA, primary antibody, and secondary antibody. Finally, protein bands were evaluated by GeneSnap using the SynGene system (SynGene, Bangalore, India) and quantified using ImageJ software.

### Luciferase Reporter Assay

The 293T cells were collected and planted in 24-well plates. After 24 h, these cells were transfected with pmirGLO- circRNA_100876-WT or pmirGLO- circRNA_ 100876-MUT plasmid and with miR-665 mimics or miR-665 NC. After 48 h, the dual-luciferase reporter assay system (Promega, Madison, WI, United States) was used to detect and evaluate the relative luciferase activity.

### Statistics

All data are shown as mean ± standard deviation (SD). Student’s *t*-test and the chi-square test were applied for data analysis using the IBM SPSS 20.0 software. All experiments were performed three times. Significant differences were considered at *P* < 0.05.

## Results

### Up-Regulation of CircRNA_100876 Predicted Poor Prognosis in GC Patients

**FIGURE 1:**
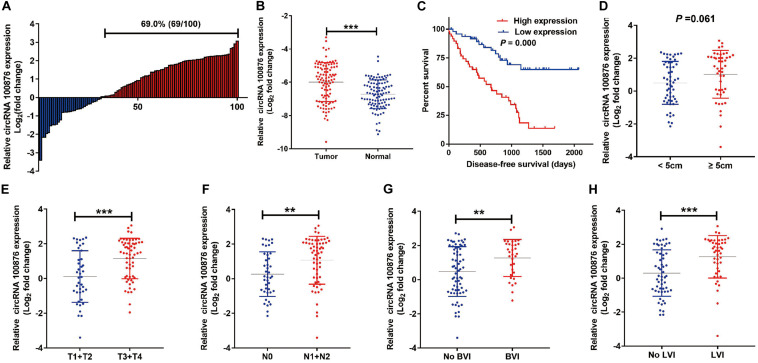
Expression level of circRNA_100876 in GC tissues. **(A)** 69% (69/100) of GC patients showed circRNA_100876 over-expression, analyzed by RT-qPCR assay. **(B)** CircRNA_100876 was up-regulated in GC tissues, analyzed by RT-qPCR assay. **(C)** Correlation between CircRNA_100876 expression and post-operative disease-free survival. **(D)** The expression of circRNA_100876 was significantly higher in patients with tumor size >5 cm. **(E)** CircRNA_100876 expression in patients with T3 or T4 tumors was increased. **(F–H)** Over-expression of circRNA_100876 was positively associated with lymph node metastasis, blood vessel infiltration, and lymphatic vessel infiltration. **P < 0.01, ***P < 0.001.

The RT-qPCR results revealed that circRNA_100876 expression was increased in 69% of all GC specimens (69/100, Figure 1A). The data were displayed after log2 logarithmic conversion processing. We found that compared with the adjacent normal tissues, circRNA_100876 expression was significantly up-regulated in GC tissues (*P* < 0.001, Figure 1B). Furthermore, the data indicated that patients with lower circRNA_100876 expression had longer disease-free survival time (*P* = 0.000, Figure 1C). In addition, circRNA_100876 expression was more likely to be highly expressed in patients with tumor size >5 cm compared to those with tumor size <5 cm (*P* = 0.061, Figure 1D). Compared to patients with T1 or T2 tumors, circRNA_100876 expression was increased in those with T3 or T4 tumors (*P* < 0.001, Figure 1E). Moreover, the patients with circRNA_100876 over-regulation were more likely to have lymph node metastasis (Figure 1F), blood vessel infiltration (Figure 1G), and lymphatic vessel infiltration (Figure 1H). Subsequently, patients were divided into high- and low-expression groups according to the median circRNA_100876 expression (Table 2). Our data confirmed that circRNA_100876 expression showed statistically significant differences with lymphatic infiltration (*P* = 0.001), tumor size (*P* = 0.016), T stage (*P* = 0.001), and lymphatic metastasis (*P* = 0.002), but not with in age (*P* = 0.687), gender (*P* = 0.680), tumor differentiation (*P* = 0.534), and vascular infiltration (*P* = 0.056). Furthermore, multivariate survival analysis (Table 3) was conducted, and circRNA_100867 expression was identified as a covariate (HR: 2.309, 95% CI: 1.129–4.716, *P* = 0.022) with T stage (HR: 2.620, 95% CI: 1.124–6.107, *P* = 0.026) and vascular invasion (HR: 2.101, 95% CI: 1.03967–4.136, *P* = 0.032).

**TABLE 2:** Correlation between circRNA_100876 expression and the clinicopathologic characteristics of patients with gastric cancer.

Clinical parameter		CircRNA_100876		*P*-value
		Low expression (*n* = 50)	High expression (*n* = 50)	
Gender	Male	32 (51.6%)	30(48.4%)	0.680
	Female	18(47.4%)	20(52.6%)	
Age	<60	29(51.8%)	27(48.2%)	0.687
	≥60	21(47.7%)	23(52.3%)	
Differentiation	Well + moderate	20(54.1%)	17(45.9%)	0.534
	Poor + undifferentiated	30(47.6%)	33(52.4%)	
Lymphatic infiltration	Negative	36(65.5%)	19(34.5%)	0.001
	Positive	14(31.1%)	31(68.9%)	
Vascular invasion	Negative	38(56.7%)	29(43.3%)	0.056
	Positive	12(36.4%)	21(63.6%)	
Tumor size (cm)	<5	33(61.1%)	21(38.9%)	0.016
	≥5	17(37.0%)	29(63.0%)	
T stage	T1 + T2	28(70.0%)	12(30.0%)	0.001
	T3 + T4	22(36.7%)	38(63.3%)	
Lymphatic metastasis	Negative	28(68.3%)	13(31.7%)	0.002
	Positive	22(37.3%)	37(62.7%)	

**TABLE 3:** Cox analyses of recurrence-free survival in patients with gastric cancer.

Clinical parameters	Univariate				Multivariate			
	HR	95% CI		*P*	HR	95% CI		*P*
Gender (male vs. female)	1.089	0.599	1.980	0.779				
Age (<60 vs. ≥60)	0.747	0.410	1.360	0.340				
Differentiation (Well + moderate vs. Poor + undifferentiated)	1.392	0.740	2.620	0.305				
Lymphatic infiltration (Negative vs. positive)	2.757	1.520	5.000	0.001	1.429	0.653	3.123	0.372
Vascular invasion (Negative vs. positive)	3.053	1.685	5.531	0.000	2.101	1.067	4.136	0.032
Tumor size (cm) (<5.0 vs. ≥5.0)	1.945	1.073	3.524	0.028	0.793	0.397	1.582	0.510
T stage (T1 + T2 vs. T3 + T4)	4.115	1.914	8.847	0.000	2.620	1.124	6.107	0.026
Lymphatic metastasis (Negative vs. positive)	2.305	1.224	4.341	0.010	0.970	0.402	2.338	0.946
CircRNA_100876 expression (Low vs. High)	3.779	1.971	7.244	0.000	2.308	1.129	4.716	0.022

### Down-Regulation of CircRNA_100876 Suppressed GC Cells’ Proliferation and Increased GC Cells’ Apoptosis *in vitro*

**FIGURE 2:**
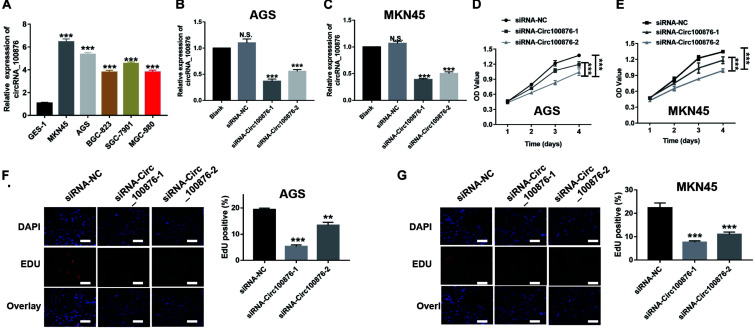
Down-regulation of circRNA_100876 suppressed GC cell proliferation. **(A)** CircRNA_100876 was over-expressed in GC cells, especially AGS and MKN45, analyzed by RT-qPCR assay. **(B,C)** The knockdown efficiency of circRNA_100876 in AGS and MKN45 cells using circRNA_100876-specific siRNAs, analyzed by RT-qPCR assay. **(D,E)** CircRNA_100876 silencing decreased AGS and MKN45 cell growth, analyzed by CCK-8 assay. **(F,G)** Down-regulation of circRNA_100876 inhibited cellular DNA replication in AGS and MKN45 cells, analyzed by EDU assay. **P < 0.05, **P < 0.01, ***P < 0.001.

**FIGURE 3:**
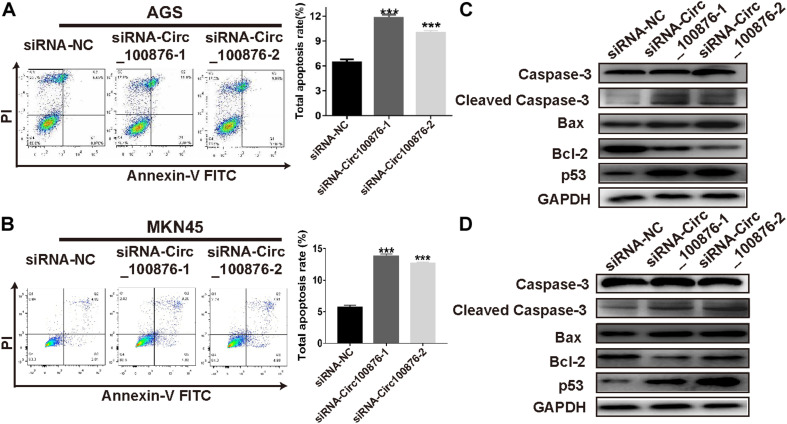
Down-regulation of circRNA_100876 increased apoptosis in GC. **(A)** Images of cell apoptosis (left) and statistical data of the apoptotic rates of AGS cells (right), analyzed by flow cytometry assay. **(B)** Images of cell apoptosis (left) and statistical data of the apoptotic rates of MKN45 cells (right), analyzed by flow cytometry assay. **(C,D)** Knockdown of circRNA_100876 increased apoptosis-related proteins, such as caspase-3, cleaved caspase-3, Bax, and P53, while the inhibition of apoptosis-related protein bcl-2 was decreased in AGS and MKN45 cells, analyzed by western blotting. ***P < 0.001.

RT-qPCR assays indicated that circRNA_100876 expression was up-regulated in GC cell lines, especially AGS and MKN45, compared with the normal gastric epithelial cell line GES-1 (Figure 2A). Subsequently, we knocked down the expression of circRNA_100876 in AGS and MKN45 cells (Figures 2B,C). From the CCK-8 assays, we found that detected that circRNA_100876 down-regulated AGS and MKN45 GC cells showed lower OD values, compared to control groups, indicating that down-regulation of circRNA_100876 could significantly suppress the proliferation ability of GC cells (Figures 2D,E). Similarly, EDU assay results revealed that circRNA_100876 knockdown could clearly reduce the proportion of EDU-positive cells in both AGS and MKN45 cell lines (Figures 2F,G). The flow cytometry results showed that circRNA_100876 knockdown increased the ratio of apoptotic AGS and MKN45 cells compared with the control groups (*P* < 0.001, Figures 3A,B). In addition, western blotting verified the increase in apoptosis-related proteins, such as cleaved caspase-3, Bax, and P53, while the inhibition of apoptosis-related protein Bcl-2 decreased (Figures 3C,D).

### Down-Regulation of CircRNA_100876 Inhibited the Migration and Invasion Abilities of GC Cells Through Suppressing the EMT Pathway

**FIGURE 4:**
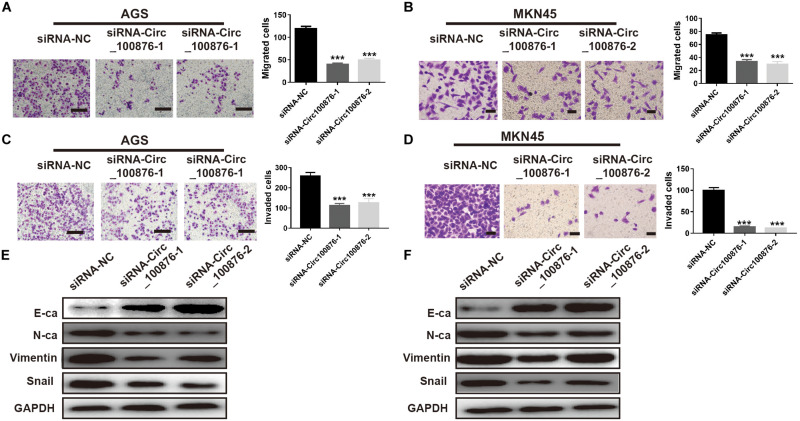
Down-regulation of circRNA_100876 suppressed migration and invasion by inhibiting epithelial–mesenchymal transition (EMT). **(A,B)** The number of migrated cells was significantly reduced in AGS **(A)** and MKN45 **(B)** cell lines according to Transwell migration assay. **(C,D)** The number of invaded cells was significantly reduced in AGS **(C)** and MKN45 **(D)** cell lines according to Transwell invasion assay. **(E,F)** The expression of the epithelial marker E-cadherin (E-ca) was up-regulated and the mesenchymal marker N-cadherin (N-ca) was down-regulated after over-expression of circRNA_100876 in AGS **(E)** and MKN45 **(F)** cell lines, analyzed by western blotting. *** *P* < 0.001.

Based on the results regarding the relationship between circRNA_100876 and clinicopathologic parameters, we have concluded that circRNA_100876 up-regulation was always accompanied with tumor invasion, lymph node metastasis, and vascular invasion. Consequently, we speculated that circRNA_100876 over-expression in GC cells might be related to migration and invasion. The Transwell assay results confirmed that the metastatic potential of GC cells was significantly limited after the depletion of circRNA_100876 (Figures 4A–D). To explore the molecular mechanism through which circRNA_100876 regulates the migration and invasion abilities of GC cells, we further detected the EMT progression of GC cells. The western blot results demonstrated an increase in proteins characteristic of an epithelial-like phenotype (E-cadherin) and a decrease in those of a mesenchymal phenotype (N-cadherin, vimentin, and snail protein) following circRNA_100876 down-regulation (Figures 4E,F). Taken together, these data suggested that the down-regulation of circRNA_100876 inhibited migration and invasion by suppressing the EMT pathway in GC.

### CircRNA_100876 Acts as a ceRNA for miR-665 to Modulate YAP1 Expression in GC Cells

**FIGURE 5:**
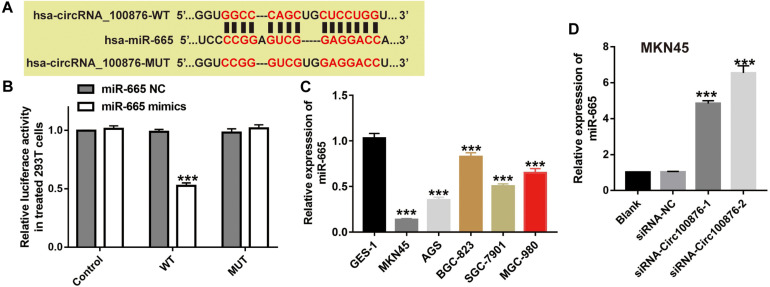
MiR-665 was a target of circRNA_100876. **(A)** The binding site of miR-665 and circRNA_100876. **(B)** Luciferase reporter assay was performed to validate these predictions. ***P < 0.001. **(C)** Expression levels of miR-665 in GC cell lines, analyzed by RT-qPCR assay. ***P < 0.001. **(D)** Expression levels of miR-665 after transfection with siRNA- circRNA_100876, analyzed by RT-qPCR assay. ***P < 0.001.

Many studies have uncovered that circRNAs may act as a ceRNA by sequestering miRNAs. To further study the potential downstream miRNA of circRNA_100876, online software program starBase v3.0^1^ was used to predict and showed that five miRNAs (miR-665, miR-652-5p, miR-922, miR-466, and miR-4739) were potential targeting miRNA of circRNA_100876. Then, luciferase activity assay was carried out to confirm the direct binding relationship between circRNA_100876 and these five potential miRNAs. Our results showed that these five miRNA mimics could reduce the luciferase activities of pmirGLO_circRNA_100876-wt, while the miR-665 mimics had the most significant inhibitory effect (Figure 5B and Supplementary Figure S1). More importantly, the binding sites between miR-665 and circRNA_100876 were shown in Figure 5A. We also found that miR-665 expression in GC cell lines was significantly lower than that in GES-1 (Figure 5C), whereas the inhibition of circRNA_100876 could significantly increase miR-665 expression (Figure 5D).

**FIGURE 6:**
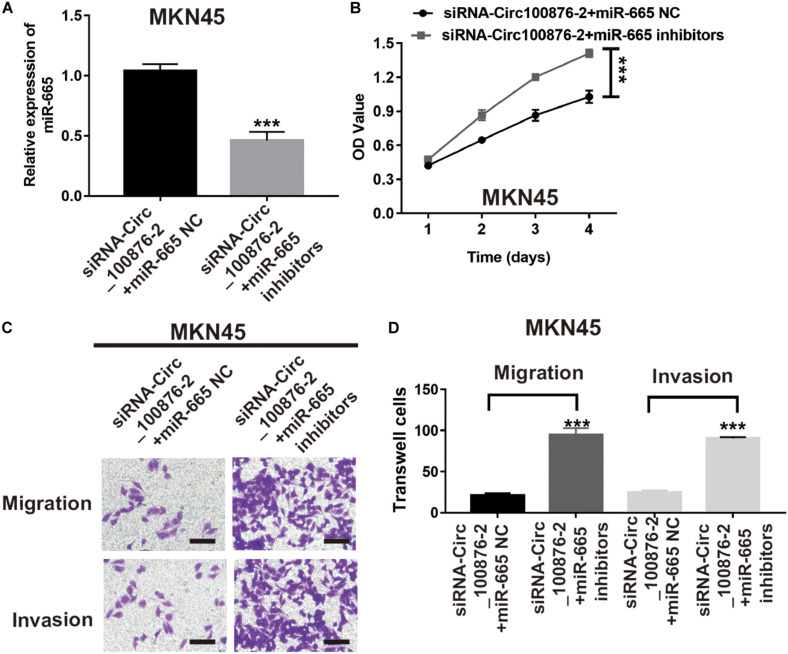
MiR-665 inhibitor prevented the effects of circRNA_100876 knockdown on MKN45 cells. **(A)** Transfection efficiency was confirmed after transfecting the miR-665 inhibitor or control inhibitor in siRNA-circRNA_100876-transfected MKN45 cells, analyzed by RT-qPCR assay. ***P < 0.001. **(B)** MKN45 cell proliferation was measured by CCK-8 assay. ***P < 0.001. **(C,D)** MKN45 cell migration and invasion was assessed by Transwell assay. ***P < 0.001.

To further verify the biological function of miR-665, we downregulated miR-665’s expression in siRNA-circ_100876-2 MKN45 cells (Figure 6A), and then CCK-8 and Transwell assays were performed to show that proliferation rates and migration and invasion abilities were significantly restored (Figures 6B–D).

**FIGURE 7:**
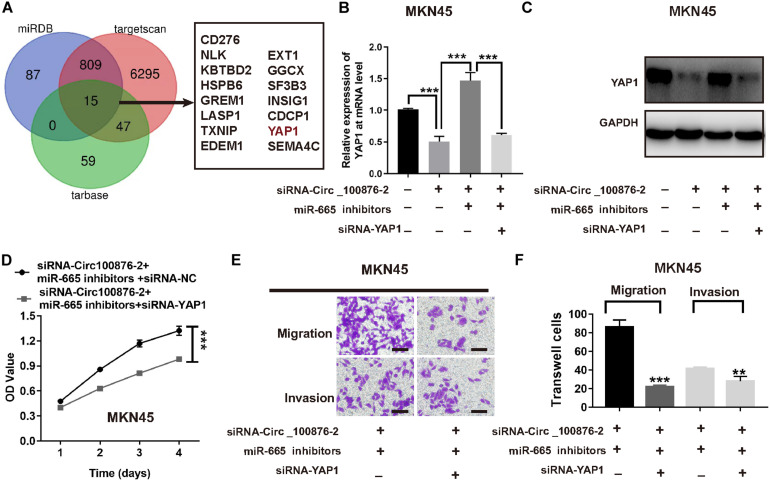
Down-regulation of YAP1 abolished the effects of miR-665 on MKN45 cells. **(A)** Venn diagram of common genes from 3 publicly available databases, including targetscan, miRDB, and starBase, was used to identify YAP1 as a target of miR-665. **(B)** Relative expression of YAP1 at mRNA levels, after transfection with siRNA-circRNA_100876 and siRNA-circRNA_100876 + miR-665 inhibitor, analyzed by RT-qPCR assay. ***P < 0.001. **(C)** Relative expression of YAP1 protein, after transfection with siRNA-circRNA_100876 and siRNA-circRNA_100876 + miR-665 inhibitor, analyzed by Western blotting assay. ***P < 0.001. **(D)** Cell proliferation of MKN54 cells transfected with siRNA-circRNA_100876 + miR-665 inhibitor was measured using CCK-8 assay. ***P < 0.001. **(E,F)** Cell migration and invasion of MKN45 cells transfected with siRNA-circRNA_100876 + miR-665 inhibitor was assessed using Transwell assay. ***P < 0.001, **P < 0.01.

In order to further investigate the ceRNA network mechanism in GC, three online bioinformatics databases (including miRDB, targetscan Human 7.2 and miRtarbase) were applied to predict the potential target genes of miR-665, then we generated Venn diagram by online webtool^2^ to visualize the intersecting genes between the results of three databases. Using a Venn diagram, we found 15 genes that were predicted by the three databases; of these, we focused on YAP1, a gene that has been reported as involved in carcinoma development, including GC (Figure 7A). Next, we verified their expression levels in the GEPIA database^3^, as YAP1 were shown to be up-regulated in GC, while its expression was highly related with GC patients’ survival time (Supplementary Figures S2A,B). Therefore, we supposed YAP1 might be the downstream of miR-665. Furthermore, RT-qPCR and western blot analysis consistently demonstrated that the inhibition of circ_100876 significantly suppressed YAP1 expression at mRNA and protein levels; however, this result could be reversed by an miR-665 inhibitor in MKN45 cells (Figures 7B,C). To verify the biological function of YAP1, we knocked down its expression in MKN45 cell lines transfected with siRNA_circ_100876 and miR-665 inhibitor (Figures 7B,C). Interestingly, both CCK-8 and Transwell assays showed that proliferation, migration and invasion abilities were significantly restored (Figures 7D–F).

## Discussion

Accumulating evidence has clarified that circRNAs play a vital role in cancer biology (Mirzaei et al., 2016; Meng et al., 2017). Abnormal expression of circRNA_100876 has been identified in non-small-cell lung cancer and esophageal squamous cell carcinoma. However, circRNA_100876 expression in GC remains unknown and is thus of clinical significance. Our data showed that circRNA_100876 were highly expressed in GC tissues compared with the corresponding non-cancerous tissues; furthermore, we determined that higher circRNA_100876 expression was clearly associated with clinical T and N staging and poor prognosis for GC patients.

Emerging findings have elucidated that thousands of circRNAs are abnormally expressed in tumors, while these specific circRNAs might promote tumor progression by sequestering miRNAs or other molecules to regulate gene expression. In recent years, increasing evidence indicates that circRNAs serve as diagnostic biomarkers in GC. For example, hsa_circ_0000190 has been shown to be down-regulated in GC patients, and this circRNA has better sensitivity and specificity in predicting the prognosis of GC patients compared to the classical GC biomarkers CEA and CA19-9 (Chen et al., 2017). CircRNA_100876 is a novel circRNA that is up-regulated in GC and holds great potential as a biomarker to predict disease progression and prognosis.

To evaluate the functions of circRNA_100876, we analyzed circRNA_100876 knockdown cells and found that the down-regulation of circRNA_100876 could inhibit cell proliferation and induce apoptosis of the cancer cell lines, indicating that circRNA_100876 was involved in GC progression. Moreover, circRNA_100876 down-regulation could clearly inhibit the migration and infiltration abilities of GC cells, suggesting that high expression of circRNA_100876 in GC tissues could promote the metastatic ability of tumor cells. In other words, circRNA_100876 is highly expressed in GC patients and promotes distant metastasis and poor prognosis.

Epithelial-mesenchymal transition is very common in the metastasis of many tumors (Nieto, 2013), and previous studies have confirmed that numerous circRNAs affect the EMT process (Conn et al., 2015). The major event of the EMT process occurs when epithelial cells become disconnected and lose their polarity and adhesion abilities; the epithelial cells then transform into mesenchymal-like cells, which enhances their motor ability. The signature change in this process is the increase in mesenchymal markers N-cadherin, vimentin, and Snail and the decrease in epithelial marker E-cadherin (Yang et al., 2018; Dongre and Weinberg, 2019). Western blotting demonstrated that after knockdown of circRNA_100876, E-cadherin increased significantly, while N-cadherin, Vimentin, and Snail protein levels decreased. Therefore, we can conclude that circRNA_100876 promotes the EMT process of GC, thus leading to tumor metastasis.

In the past decade, numerous studies have confirmed that circRNAs act as ceRNAs to sequester miRNAs and affect their transcriptional regulation. Using bioinformatics methods, we found that miR-665 and circRNA_100876 have potential binding sites. Additionally, miR-665 has been demonstrated to participate in the development of various carcinomas, such as ovarian cancer, pancreatic cancer, and colorectal cancer (Liu et al., 2018; Zhou et al., 2018; Ouyang et al., 2019). For example, Liu J. et al. indicated that miR-665 suppresses the growth and migration via regulating HOXA10 in ovarian cancer cells (Liu et al., 2018). However, the role of miR-665 in GC remains unclear. In current study, our results showed that the addition of miR-665 inhibitors could reverse the anti-tumor effects induced by the down-regulation of the circRNA-100876 in GC. Next, miRDB, Targetscan, and starBase were used to predict the target genes of miR-665. Among all target genes, we focused on YAP1. As we knew, YAP1 has been confirmed to be over-expressed in GC and associated with the progression, lymph node metastasis, and poor prognosis of GC (Hu et al., 2014). In our study, we showed that YAP1 was a target gene of miR-665. Similarly, our results revealed that the suppression of YAP1 caused decreases in cell proliferation, migration and invasion. These results suggest that miR-665 exerted its tumor suppressor effects by targeting YAP1 in GC.

## Conclusion

**Scheme 1:**
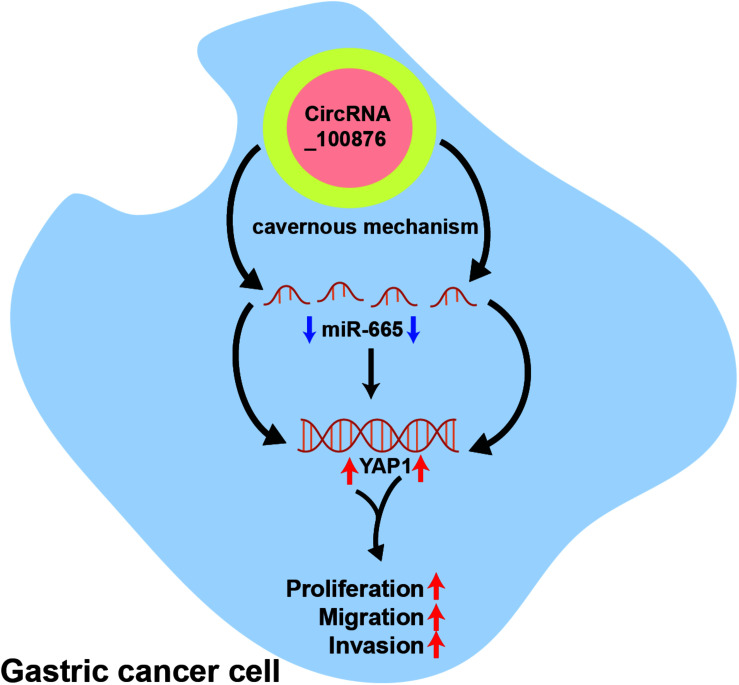
CircRNA_100876 promotes GC progression through regulating miR-665/YAP1 axis.

We have demonstrated that circRNA_100876 is over-expressed and promotes tumor growth and metastasis by sequestering miR-665 in GC. Furthermore, the inhibition of miR-665 expression induced YAP1 to promote tumor proliferation, invasion, and metastasis. However, there are still some limitations in our research. Firstly, the circRNA_100876-overexpressed cell models should be constructed for fully confirming the biological function of circRNA_100876 in the future research. Secondly, the *in vivo* experiments need further explored. In short, circRNA_100876 is up-regulated in GC and promotes its growth and metastasis through miR-665/YAP1 signaling (Scheme 1).

## Data Availability Statement

The original contributions presented in the study are included in the article/Supplementary Material, further inquiries can be directed to the corresponding author/s.

## Ethics Statement

The studies involving human participants were reviewed and approved by the Ethics Committee of the First Affiliated Hospital of Shantou University Medical College.

## Author Contributions

XL, CH, and ZC carried out most of the experiments, and HW completed the bioinformatics analysis and wrote the manuscript. HW and YZ proposed the idea and supported the project. All authors read and approved the final manuscript.

## Conflict of Interest

The authors declare that the research was conducted in the absence of any commercial or financial relationships that could be construed as a potential conflict of interest.

## Footnotes

1 http://starbase.sysu.edu.cn/

2 http://bioinformatics.psb.ugent.be/webtools/Venn/

3 http://gepia.cancer-pku.cn/

## Supplementary Material

Supplementary Figure 1 Luciferase reporter assay was performed to validate the correlation between four potential miRNAs (miR-652-5p, miR-922, miR-466, and miR-4739) and circRNA_100876. **P <* 0.05, ***P* < 0.01.

Supplementary Figure 2 Expression level of YAP1 in GC tissues based on the results of GEPIA database. **(A)** YAP1 was up-regulated in GC tissues, analyzed by RT-qPCR assay. **(B)** Correlation between YAP1 expression and GC patients’ survival time.
