# Supplementary material for: CircRNA_100876 Is Upregulated in Gastric Cancer (GC) and Promotes the GC Cells’ Growth, Migration and Invasion via miR-665/YAP1 Signaling
Source: Front Genet. 2020 Nov 11;11:546275. doi: 10.3389/fgene.2020.546275 (PMC7686782; doi:10.3389/fgene.2020.546275)
Supplement: Supplementary Figure 1 — Luciferase reporter assay was performed to validate the correlation between four potential miRNAs (miR-652-5p, miR-922, miR-466, and miR-4739) and circRNA_100876. *P < 0.05, **P < 0.01. [file Data_Sheet_1.docx]

*Supplementary Material*

***Results***


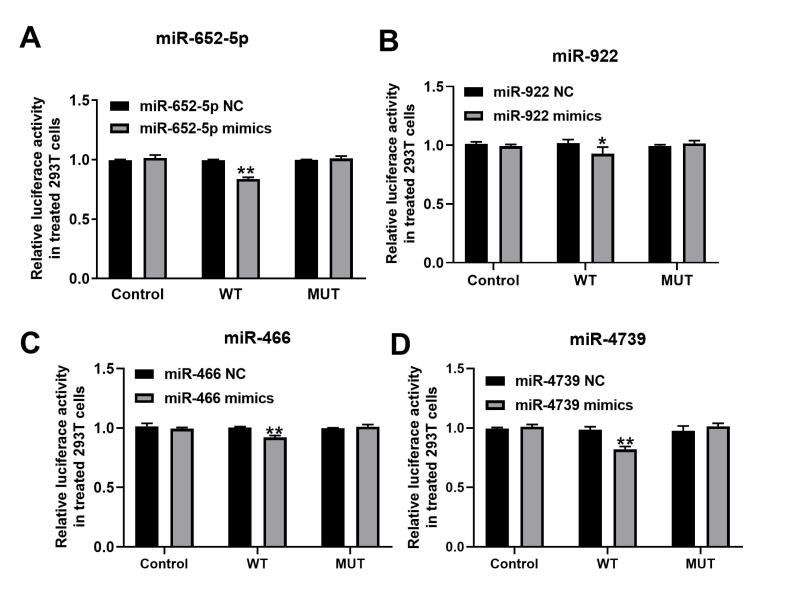


***Figure S1. Luciferase reporter assay was performed to validate the correlation between four potential miRNAs (miR-652-5p, miR-922, miR-466 and miR-4739) and circRNA_100876.*** ****P<0.05, **P<0.01.***


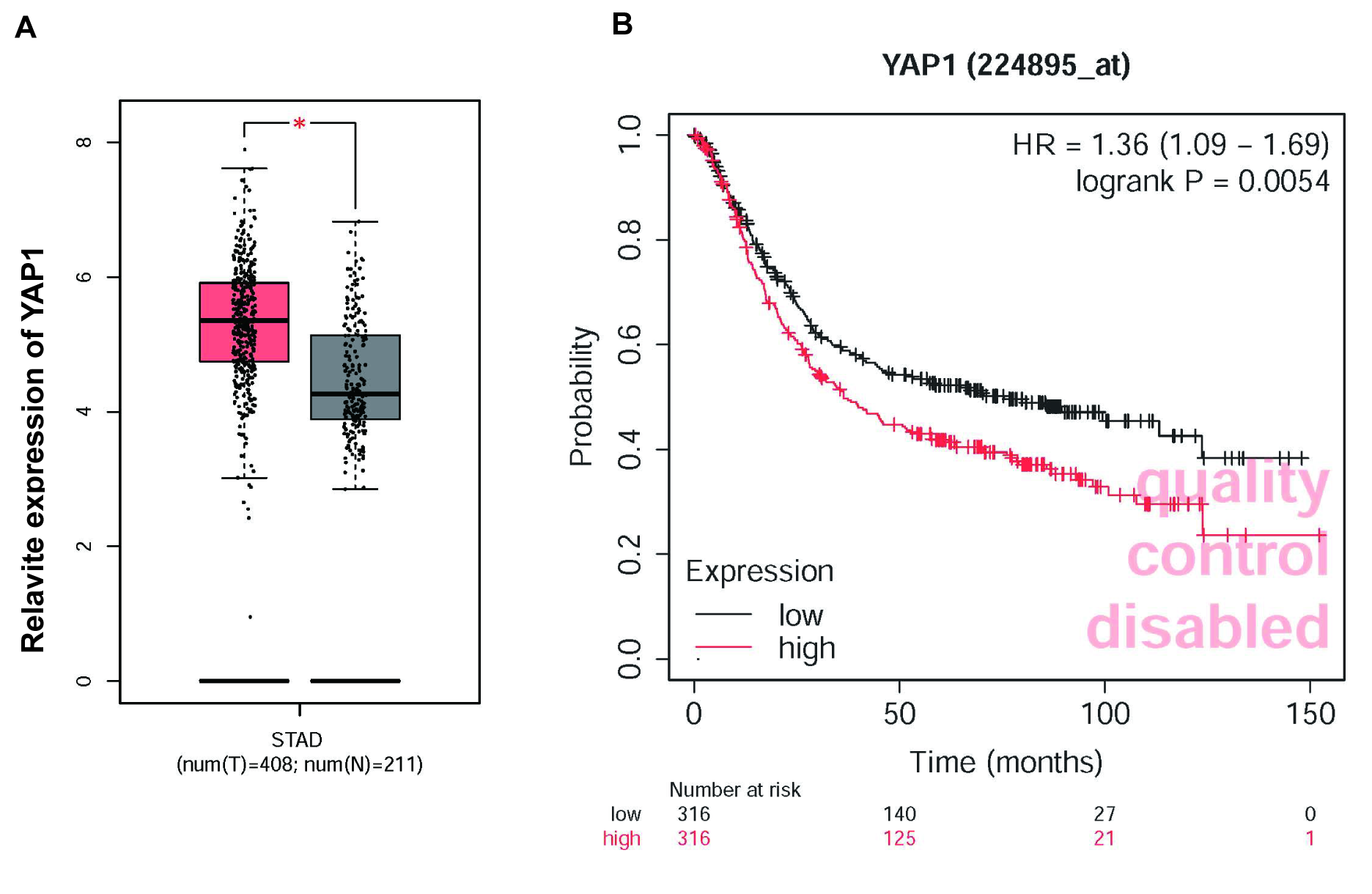


***Figure S2. Expression level of YAP1 in GC tissues based on the results of GEPIA database.*** *(A) YAP1 was up-regulated in GC tissues, analyzed by RT-qPCR assay. (B) Correlation between YAP1 expression and GC patients’ survival time.*P<0.05.*
